# A multi-etiological model of childhood obesity: a new biobehavioral perspective for prevention?

**DOI:** 10.1186/s13052-019-0762-3

**Published:** 2019-12-27

**Authors:** Giuliana Valerio, Sergio Bernasconi

**Affiliations:** 1 Department of Movement Sciences and Wellbeing, University of Naples Parthenope, via Medina, 40, 80133 Naples, Italy; 2 Microbiome Research Hub, University of Parma, Parma, Italy

**Keywords:** Behaviour, Obesity, Prevention

## Abstract

Current prevention strategies focusing only around the energy balance model have been found insufficient to tackle the childhood obesity epidemic. Originating from the paper by Baranowski et al., recently published in Current Nutrition Report, this Commentary is aimed at discussing the complex etiology of obesity, on the ground of new biological models, which open a novel biobehavioral perspective of prevention.

## Background

Obesity represents a major health problem that needs to be recognized and prevented early in the life [1]. Childhood obesity has reached epidemic proportions in the last decades, although a trend to stabilization has been observed. Children and adolescents who are obese, particularly those who are severely obese, present physical comorbidities previously observed only in adults, and are much more likely to be obese as adults compared to their normal weight peers. Cardiometabolic risk factors have an earlier onset and a prolonged effect in obese children, and the resulting complications, such as cardiovascular disease, type 2 diabetes or steatohepatitis can be more severe. Mortality rates increase dramatically with increasing body mass index (BMI), and the lifespan of individuals with obesity can be up to 8–10 years shorter than normal weight people [2].

Reducing BMI and maintaining weight loss is challenging, since the components of energy balance compensate weight loss by counteracting the negative energy balance [3]. On the contrary, the body system does not react to the weight that has not yet been gained. Therefore, prevention of childhood obesity is better than cure, and the need for more effective strategies to prevent obesity is of primary importance. Halting the global obesity rates in school-aged children, adolescents and adults is one of the nine targets to be attained in 2025 by the Global Action Plan for the prevention and control of non-communicable diseases of the World Health Organization [4].

The aim of this Commentary is to discuss the complex etiology of obesity, on the ground of new biological models, which open a novel biobehavioral perspective of prevention.

Childhood obesity is the consequence of an interaction among a complex set of environmental, genetic, and psychosocial determinants that lead to excessive food intake and insufficient physical activity. Energy dense, nutrient-poor foods and beverages are now more affordable and accessible. Industrialization, urbanization and technological progress caused a decline in physical activity and increase of sedentary behaviors. Therefore, the interventions for preventing overweight and obesity are predominantly aimed to promote healthy eating and an active lifestyle, increasing the daily level of physical activity and decreasing the duration of screen time.

## Main text

Behavioral changes are the primary tools for modifying lifestyle and preventing obesity. However, current prevention strategies for childhood overweight and obesity show very small effects and high heterogeneity. The most recent Cochrane review on randomized controlled trials of diet and/or physical activity interventions for preventing obesity in children concluded that children aged 0–5 years were the most responsive to diet combined with physical activity interventions, while interventions focused only on physical activity were more promising in children aged 6 to 12 years, and at lesser extent in adolescents aged 13 to 18 years [5].

Why is it so hard to tackle obesity in childhood? The environment variously contributes to the unhealthy eating practices and sedentary lifestyle, therefore the classical strategies relying solely on the individual responsibility are not effective. A change in behaviour aimed at disrupting positive energy balance requires environment-oriented measures to make healthier choices more available and accessible. Indeed, the most effective interventions to prevent childhood obesity should consider different settings and multiple strategies, with a life-course approach [1]. A comparative review of the effectiveness of childhood obesity prevention programs ascribed high strength of evidence to school-based diet and physical activity interventions that included home and community components, while moderate strength of evidence was found for school based interventions associated to either home or community setting [6].

Obesity is a complex and multi-factorial disease. Therefore, prevention focused only on the model of energy balance does not seem to be completely effective. This model indeed, as recently pointed out by Baranowski et al. [7], does not take into consideration factors that are still insufficiently studied and that can contribute to the pathogenesis of obesity.

New prevention strategies should therefore consider a more comprehensive biological model, in which specific risk factors are better identified allowing to intervene with more individualized preventive programs. In addition to genetic factors, three candidate etiologies were discussed by Baranowski et al. [7]: infectobesity, gut microbiome, and circadian rhythms.

Specific viruses have been identified that likely cause some cases of obesity. For instance, herpes viruses and cytomegalovirus have been associated with obesity traits in childhood [8]. Individuals infected by adenovirus-36 have an odds ratio of 2.0 for obesity and a standardized mean difference of 0.28 for BMI compared to people who are not infected. Adenovirus infection can reduce leptin levels, influence the signals responsible of stem cells transformation into mature adipocytes or alter fat and carbohydrate metabolism, leading to hyperplasia and hypertrophy of adipocytes.

The potential link between microbioma and obesity represents another fascinating field for prevention [9, 10]. Imbalances in gut microorganisms, such as the increase of the Firmicutes/Bacteroidetes ratio, might play an important role in the pathogenesis of obesity, as reported both in mice and humans [11]. Low bacterial prevalence characterized individuals with overall adiposity, insulin resistance and dyslipidemia [12]. Diet might also influence the microbiota composition and function. Fat- or carbohydrate-restricted low-calorie diet increased Bacteroidetes while reduced Firmicutes in obese adults, [11]. Research is ongoing on the determinants of neonatal microbiome, such as cesarean section, early antibiotic exposure, and formula feeding, while it is still unexplored how the different aspects of diet (macronutrient distribution, dietary fiber, vegetarian diet, use of prebiotics or probiotics), physical activity levels, and other behaviors may influence the microbiome.

Several cumulating evidences in rodents and humans clearly demonstrate a strict interrelationship among the circadian system, metabolism, and behavior [13]. Circadian misalignment induced by mistimed light exposure, timing of sleep or food intake may adversely affect glucose, lipid and energy metabolism, with a high risk of obesity and diabetes. Dietary nutrients can influence circadian rhythms at both molecular and behavioral levels, but the time and content of meals (e.g., macronutrients, caffeine, alcohol) need to be better understood [14].

In the last few years, in addition to the above mentioned factors, numerous studies on both animal models and humans have highlighted the role of endocrine disruptors in adipogenesis and related complications, such as insulin resistance [15].

The solution to the obesity epidemy is not simply achieved by reducing calories and increasing physical activity. The complex interrelationship between genetic factors on one hand, and diet, physical activity, sedentary behaviors, sleep, circadian rhythmicity, environment, gut microbiome on the other, indicate that failure of preventive or treatment programs cannot be simply ascribed to lack of self-control or scarce assertiveness of individuals.

Next generation preventive and treatment strategies should be driven by better understanding of how energy balance is achieved by the body, in order to identify those individuals at highest risk for diverse causes.

Baranowski et al. proposed a new biobehavioral perspective of obesity prevention. If the infectobesity theory will be proven to cause adiposity in substantially large numbers of people, then obesity prevention programs would focus on sanitation and hygiene practices to avoid the contact or on the use of a vaccine, if available. On the other hand, the modulation of specific bacteria or bacterial metabolic activities, as suggested by the microbioma hypothesis, or the alignment of eating and sleep/wake patterns with endogenous rhythms, as suggested by the studies of circadian cycle, may be the target of promising strategies for obesity prevention.

## Conclusions

More globally, it may be useful to attempt new approaches to childhood obesity research, following the model of other pathological conditions, such as systemic lupus erythematosus [16]. In fact, the modern technology allows us to evaluate the predisposing factors on the genetic ground and, through the “omics” (metabolomic, proteinomic), to reconstruct the exposome. In this way, it is possible to better understand which are the agents involved in the pathogenesis of obesity in the single individual or subgroups of a population, and thus allow primary or secondary prevention.

## Data Availability

Not applicable.
